# PredHSP: Sequence Based Proteome-Wide Heat Shock Protein Prediction and Classification Tool to Unlock the Stress Biology

**DOI:** 10.1371/journal.pone.0155872

**Published:** 2016-05-19

**Authors:** Ravindra Kumar, Bandana Kumari, Manish Kumar

**Affiliations:** Department of Biophysics, University of Delhi South Campus, New Delhi, India; Russian Academy of Sciences, Institute for Biological Instrumentation, RUSSIAN FEDERATION

## Abstract

Heat shock proteins are chaperonic proteins, which are present in every domain of life. They play a crucial role in folding/unfolding of proteins, their sorting and assembly into multi-protein complex, cell cycle control and also protect the cell during stress. Considering the fact that no web-based predictor is available for simultaneous prediction and classification of HSPs, it is imperative to develop a method, which can predict and classify them efficiently. In this study, we have developed coupled amino acid composition and support vector machine based two-tier method, PredHSP that identifies heat shock proteins (1^st^ tier) and classifies it to different families (at 2^nd^ tier). At 1^st^ tier, we achieved maximum accuracy 76.66% with MCC 0.43, while at 2^nd^ tier we achieved maximum accuracy 96.36% with MCC 0.87 for HSP20, 91.91% with MCC 0.83 for HSP40, 95.96% with MCC 0.72 for HSP60, 91.87% with MCC 0.71 for HSP70, 98.43% with MCC 0.70 for HSP90 and 97.48% with MCC 0.71 for HSP100. We have also developed a webserver, as well as standalone package for the use of scientific community, which can be accessed at http://14.139.227.92/mkumar/predhsp/index.html.

## Introduction

Heat shock proteins (HSPs) are stress-induced proteins, ubiquitously found in all organisms, ranging from bacteria to human. They are one of the largest groups of molecular chaperones that assist in correct folding of partially folded or denatured proteins. Depending on the molecular weight and core functions, six major families of HSPs have been reported: (i) HSP20 or small heat shock proteins (sHsp), (ii) Hsp40 or J-class proteins, (iii) Hsp60 or chaperonins, (iv) Hsp70, (v) Hsp90, and (vi) Hsp100/ClpB protein [1, 2]. HSPs play a vital role in cellular stress response against unfavourable environmental condition like physical (temperature elevation) or chemical (increase or decrease in pH, salinity, or oxygen concentration). To protect the cell from the destructive effects of stress, HSPs promote attainment of functional conformation of partially denatured proteins [3]. The activities of stress proteins are not limited to the chaperoning of other proteins but also includes other functions, like, modulation of their own synthesis [4], regulation of the stress kinase JNK [5], participation in signal transduction pathways [6] and in rRNA processing [7]. Due to the wide range of functional activities, malfunctioning of HSPs leads to a number of life-threatening diseases that includes Parkinson’s disease [8], Alzheimer’s disease [9], cardiovascular diseases [10] and cancer [11].

Due to availability of rapid and relatively inexpensive genome sequencing technologies, a large number of protein sequences are continuously added into the databases. A major fraction of these sequences are not annotated. Considering the time and resources involved in experimental annotations, these sequences are very unlikely to be annotated in the near future. This makes computational pipelines an ideal choice for annotation due to their inexpensive and high throughput nature. Considering the importance of HSPs in cellular metabolism and number of un-annotated sequences in the databases that might be HSPs, development of computational method to identify HSPs and classify their family only on the basis of primary protein sequence will have a far reaching effect. Two attempts have already been made by (i) Feng et al. [1] and (ii) Ahmad et al. [12] regarding HSP protein annotation but only for their classification into different HSP families. But methods have following shortcomings; *(i)* they do not have provision for classifying HSP family without first verifying that query proteins is HSP or not, *(ii)* method developed by Ahmad et al. [12], does not provide any web based tool or standalone software for the prediction purpose.

Here, we describe PredHSP to address the shortcomings of existing methods. PredHSP is capable to predict HSP and also its different families. It is based on coupled amino acid composition (CAA) based sequence encapsulation as input and support vector machine (SVM) as the prediction machine.

## Materials and Methods

### Data Source

#### Training Dataset

To develop PredHSP, we used the same dataset recently reported to develop iHSP-PseRAAAC [1]. The dataset was originally derived from HSPIR database [2]. Further they removed the sequences having ≥40% sequence similarity within the same subset by using CD-HIT [13], and obtained 2225 sequences from different HSP families (Table 1). 10000 non-HSP sequences were also randomly picked from SwissProt keeping in mind that no two sequences are homologous. During training HSP sequences were used as positive dataset while non-HSP sequences were used as negative dataset.

**Table 1:** Protein distribution in training dataset.

HSP Family	Description	Number of proteins
HSP20	sHSP/Small HSP	357
HSP40	DnaJ-class proteins	1279
HSP60	GroEL/ES or chaperonins	163
HSP70	DnaK/chaperones	283
HSP90	Chaperonines	58
HSP100	High Molecular Weight HSP	85
**Total**		**2225**
**Non-HSP**	**—**	**10,000**

#### Independent Dataset

We built two independent datasets having sequences of different HSP families (Table 2): *i)* an HGNC dataset [14] having 95 human HSPs (collected from HUGO Gene Nomenclature Committee (HGNC) database), *ii)* a mixed dataset of 55 rice HSPs. For mixed dataset HSPs reported in two different research papers were used: 31 HSPs were obtained from Wang et al [15] and 24 HSPs of single family, namely HSP70, were obtained from Sarkar et al [16].

**Table 2:** Distribution of HSPs across different families in independent datasets. HGNC dataset contains human HSPs obtained from HGNC [14] and mixed dataset contains rice HSPs obtained from Wang et al [15] and Sarkar et al [16].

HSP family	Number of Proteins		
	HGNC Dataset	Mixed Dataset	
		Wang et al	Sarkar et al
HSP20	11	14	—
HSP40	49	—	—
HSP60	14	4	—
HSP70	17	7	24
HSP90	4	3	—
HSP100	—	3	
**Total**	**95**	**31**	**24**

### Genome Wide Prediction of HSPs

We downloaded nine different proteomes from Uniprot, one was from archaea (*Methanothermobacter thermautotrophicus*), two were from prokaryotes (*Escherichia coli*, *Mycobacterium tuberculosis*) and six were from eukaryotes that included common baker yeast (*Saccharomyces cerevisiae*), plants (*Arabidopsis thaliana*, *Oryza sativa*), and animals (*Caenorhabditis elegans*, *Drosophila melanogaster*, *Homo sapiens*). Using PredHSP annotation pipeline we predicted the HSPs and annotated their family at proteome level. The total number of proteins were 1868, 4305, 3993, 6721, 31480, 37386, 26612, 22006, 70076 in *Methanothermobacter thermautotrophicus*, *Escherichia coli*, *Mycobacterium tuberculosis*, *Saccharomyces cerevisiae*, *Arabidopsis thaliana*, *Oryza sativa*, *Caenorhabditis elegans*, *Drosophila melanogaster* and *Homo sapiens* respectively.

### Prediction Schema

**Fig 1:**
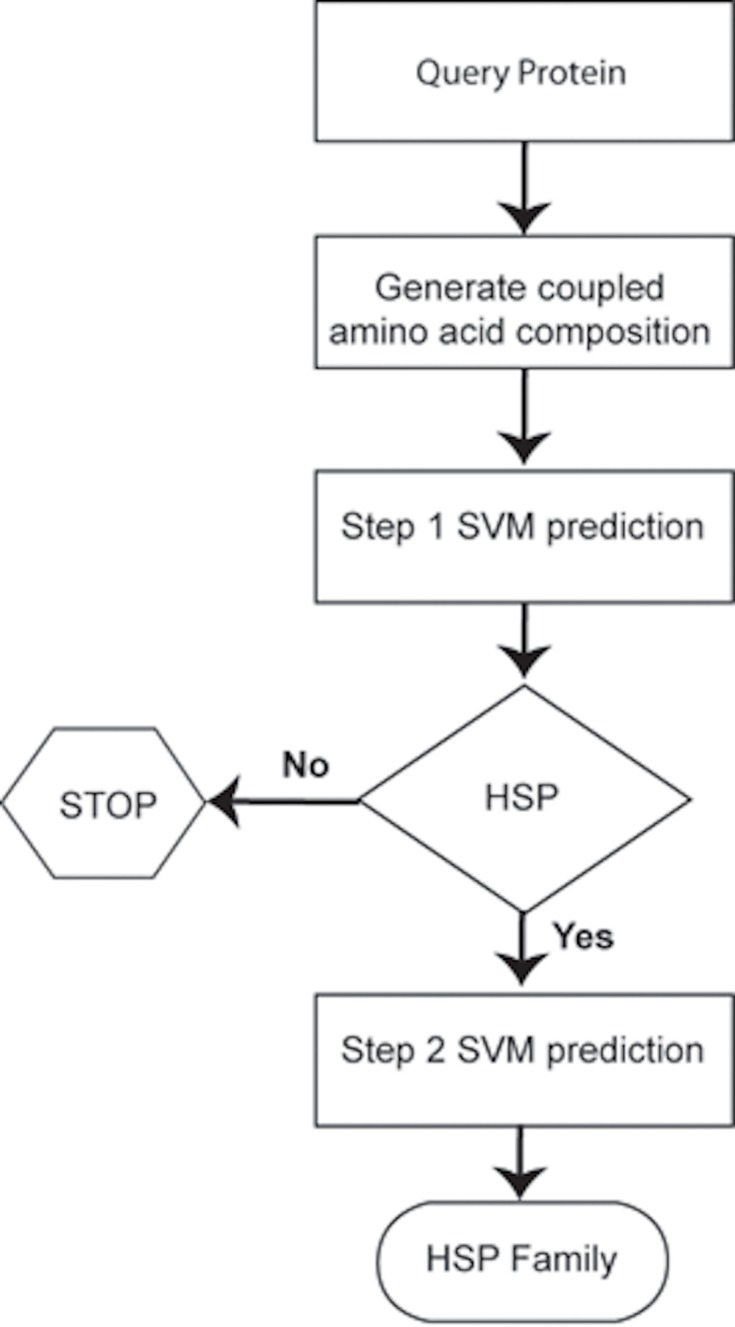
Flow chart to show the prediction schema of HSPs and its families.

Considering the heterogeneous nature of HSPs, generally multi-class classification approach is being used to predict various HSP families. Multi-class classification-based predictors assume that the input/query sequence(s) belong(s) to the same class whose sub-class is to be predicted. This assumption might work during training, which is being done on a curated data but in reality or during blind prediction, a non-class member may be used as a query protein, which may cause the wrong prediction as a class member to which it did not belong. To reduce the likelihood of wrong classification, we adopted a two-tier approach. At 1^st^ tier, non-HSPs were filtered out and only HSP sequences were passed to the 2^nd^ tier where the family was predicted (Fig 1).

### Support Vector Machine

Support vector machine is one of the popular classifiers [17] used for development of many bioinformatics prediction methods [18–20]. We used SVM_light package [21] in this work.

### SVM Model Generation

In order to develop 1^st^ tier of predictor, which can discriminate HSPs from non-HSPs, we developed the SVM model from 10,000 non-HSPs and 2,225 HSPs, which was labelled as negative and positive dataset respectively. For 2^nd^ tier, which is a multi-class classification problem, a series of binary classifiers were developed. Each classifier was capable to predict heat shock proteins of a particular family. Classifiers used for HSP class prediction were actually SVM models, trained on the HSPs only (Table 1). During training all proteins of the family, for whose prediction the SVM model was being generated, were labelled positive and proteins of remaining families were labelled negative. Same approach has been used in a number of earlier studies like prediction of sub-cellular localization [18, 22, 23], β-lactamase and its class prediction [19], G-protein coupled receptors [24], nuclear receptor protein sub-family prediction [20, 25–27].

### Cross-Validation and Performance Evaluation

Cross-validation is a way to estimate the performance of a prediction model during training. It is done on a dataset, which is not used during training. It involves partitioning of data into multiple sub-sets, performing the analysis on one sub-set (called training set), and validating the analysis on other sub-set (called testing set). The former process is called as training while the later as testing. To reduce variability in performance due to sample partition, multiple rounds of cross-validations were performed using different data partitions and the final result was obtained after averaging the results of all partitions. In the present work five-fold cross validation (FFCV) and *leave-one-out* cross validation (LOOCV), also named as jack-knife approach was used during 1^st^ and 2^nd^ tier respectively.

FFCV divides whole dataset into five sub-sets. Each sub-set consists of one-fifth of HSP and one-fifth of non-HSP. In each cycle of training four sub-sets were combined to make training set and the remaining one sub-set was used for testing. This process was repeated five times so that each sub-set was used once for testing. LOOCV partitions entire data into multiple training and test set pairs, whose number is equal to the number of sequences in dataset. In each pair, training set contains all except one sequence, while testing set contains the remaining one. During 1^st^ tier, since we had to train a large data with 12,225 sequences, FFCV approach was used. Using LOOCV on a dataset composed of a large number of sequences is time consuming as total number of training-test pairs generated during LOOCV is equal to the total number of sequences used. For the 2^nd^ tier of prediction where we had relatively small data from each HSP family, LOOCV approach of training was used. At a selected parameter, SVM model was generated using the training set and performance was evaluated on corresponding test set. On the basis of actual and predicted state, each prediction was classified into four distinct categories: true positive (TP), true negative (TN), false positive (FP), and false negative (FN). For better explanation, we describe them in context of prediction schema.

**Fig 2:**
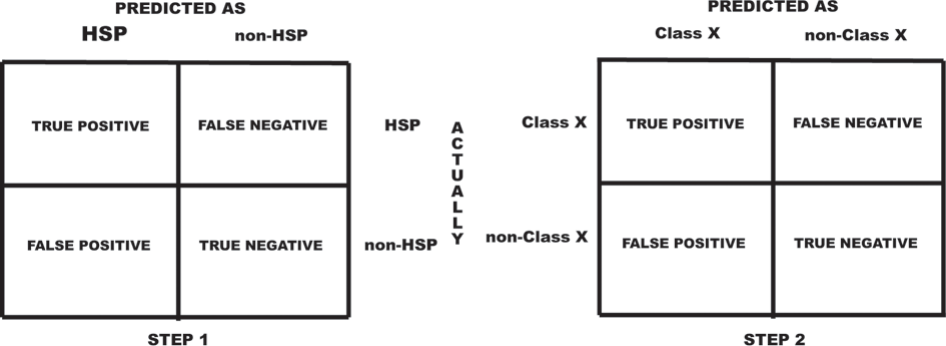
Schematic illustration of categorization of prediction into different categories.

At tier 1, TP represents the number of proteins, which are actually HSPs and also predicted as HSPs. TN represents the number of proteins which are actually non-HSPs and also predicted as non-HSPs. FP is number of non-HSPs, predicted as HSPs while FN is number of proteins which are actually HSPs but predicted as non-HSPs (Fig 2). In tier 2, since the classification was done to predict the family of a known HSP, the meaning of TP, TN, FP and FN have also changed accordingly. For a hypothetical family X, TP is the number of correctly predicted sequence that belongs to family X; TN is the number of non-family member also predicted as not a member of family X; FP is the number of sequences wrongly predicted to belong to family X while FN is the number of sequences which actually belongs to family X but predicted as non-family protein (Fig 2).

Above-mentioned four prediction indices were used to calculate three additional parameters namely, sensitivity, specificity and accuracy. A sensitivity of 100% implies that the classifier identifies all HSPs and their family correctly. Specificity of 100% means all non-HSPs and non-family members were correctly predicted. Accuracy presents overall picture and shows how well the classifier distinguishes true positives and true negatives in entire prediction. 100% accuracy denotes a perfect prediction.

$$Sensitivity=\frac{TP}{TP+FN}\times 100$$ (1)

$$Specificity=\frac{TN}{TN+FP}\times 100$$ (2)

$$Accuracy=\frac{TP+TN}{TP+FP+TN+FN}\times 100$$ (3)

Another criterion used for the prediction evaluation was Matthew’s correlation coefficient (MCC), which takes over- and under-predictions into account [28]. MCC = 1 denotes a perfect prediction, MCC = 0 indicates a completely random assignment, and MCC = -1 means a completely reverse prediction. MCC is defined as follows:

$$MCC=\frac{\left(TP\times TN)-(FP\times FN\right)}{\sqrt{\left(TP+FP)\times (TP+FN)\times (TN+FP)\times (TN+FN\right)}}$$ (4)

### Input Feature Encoding

Any SVM based prediction method requires a fixed length input. In order to extract fixed length vector from the protein sequences of different lengths, a number of encoding methods have been used to represent different forms of amino acid compositions *viz*., discrete amino acid composition (AA) [20, 29], pseudo amino acid composition (PseAA) [19, 30], coupled amino acid composition [20, 31] and split amino acid composition (SAA) [18, 32]. In this work, we used discrete amino acid composition and coupled amino acid composition to encode variable length protein sequence information into fixed length input to train SVM.

#### Discrete Amino Acid Composition

Discrete amino acid composition is the most popular and simplest way to represent a protein sequence. It is the fraction of each amino acid present in a protein sequence. Hence it encapsulates a protein sequence in a vector of 20 dimensions. It is calculated using the expression:

$$comp(i)=\frac{R_{i}}{N}\times 100$$ (5)

Where, *comp(i)* is the amino acid composition of residue type *Ri* and *N* is the total number of amino acids.

#### Coupled Amino Acids Composition

One of the main drawbacks of discrete amino acid composition is that it only uses total amino acid information but ignores the local order information of amino acids in the protein. In order to incorporate the local sequence order information along with amino acid compositions, coupled amino acid composition was also used as input. The coupled amino acid composition provides a fixed pattern length of 400. It is calculated using following expression:

$$Coupled\;AA(j)=\frac{M_{j}}{N_{coupled\;AA}}\times 100$$ (6)

Where, *Coupled AA(j) =* coupled amino acid composition of residue type M_j_; *j* = 1 to 400 and *N*_*coupled AA*_ is the total number of possible coupled amino acid composition.

## Results and Discussion

### Amino Acid Composition Analysis

In order to analyse the general trend of amino acids in heat shock proteins and in their families, we performed amino acid composition analysis using Composition Profiler [33]. Statistical significance of analysis was estimated at P-value ≤ 0.05. Composition Profiler calculates the fractional difference between the distributions of a particular amino acid (say *aa*) in two different samples (*X* and *Y*) as follows:

$$Fractional\;difference=\frac{Xaa-Yaa}{Yaa}$$ (7)

The fractional difference determines the relative enrichment/depletion of *aa* in query sample *X*, against the *aa* in background sample *Y*.

**Fig 3:**
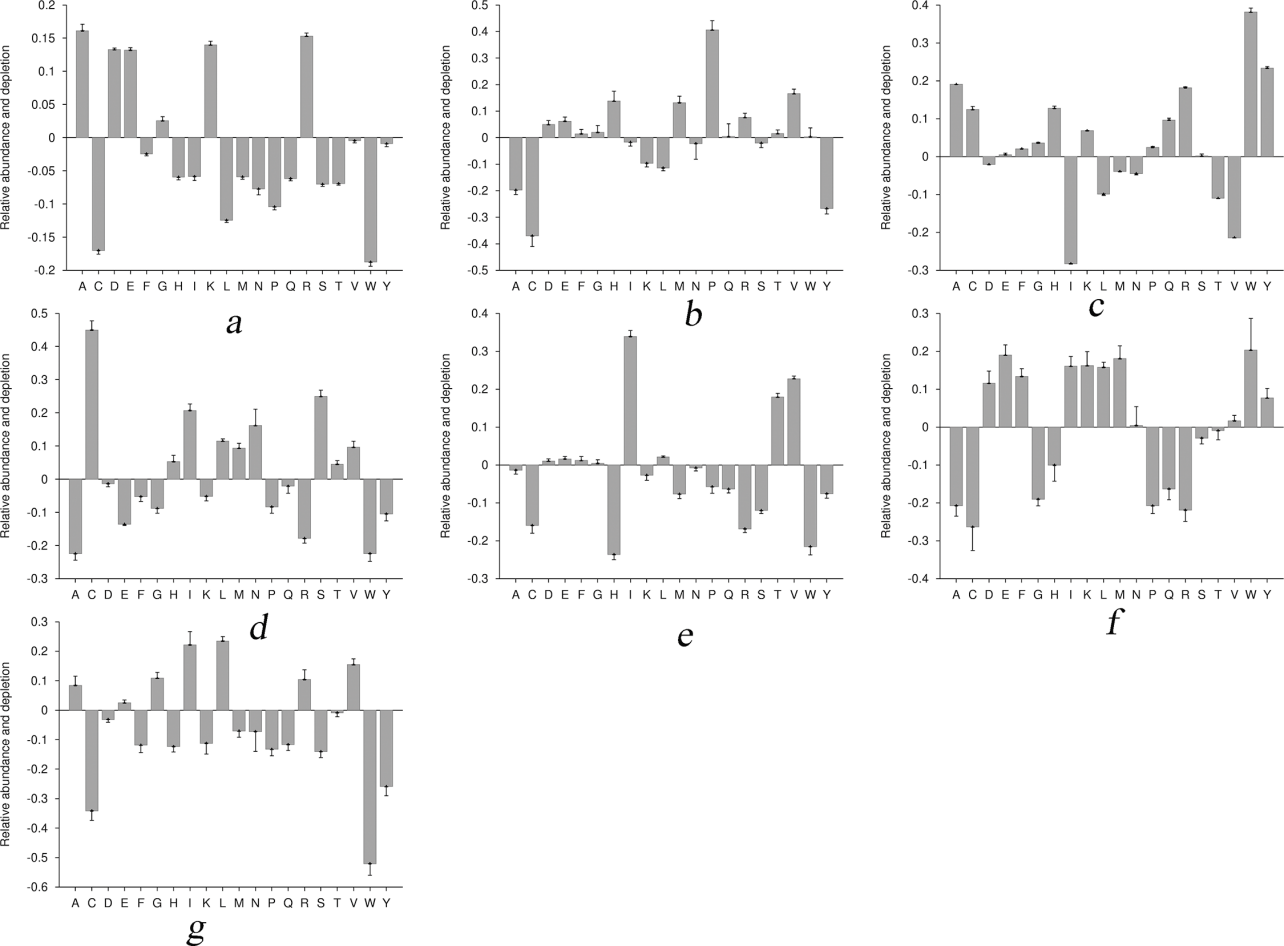
Relative enrichment and depletion of amino acids in HSP and their families with reference to non-HSP and other HSP families respectively. (3a) HSPs *vs*. Non-HSPs; (3b) HSP20 *vs*. remaining HSP family; (3c) HSP40 *vs*. remaining HSP family; (3d) HSP60 *vs*. remaining HSP family; (3e) HSP70 *vs*. remaining HSP family; (3f) HSP90 *vs*. remaining HSP family; (3g) HSP100 *vs*. remaining HSP family.

To analyse the behaviour of amino acids in heat shock proteins, we used all HSPs of the training dataset as query while all non-HSPs were used as background sample. The result shows that the HSPs were enriched with charged (both positive and negative) and polar residues but depleted of hydrophobic and aromatic residues (Fig 3A).

At 2^nd^ level i.e. at family level, one family of HSPs was used as query group and remaining all families were together used as background. For example, to analyse the amino acid enrichment and depletion pattern of HSP20, sequences belonging to HSP20 were used as query sample and remaining sequences (belonging to the HSP40, HSP60, HSP70, HSP90 and HSP100) were used as background.

In HSP20 family (Fig 3B), the distribution of negative charged residues were high while aromatic as well as hydrophobic amino acid residues was low. In HSP40 family (Fig 3C), distribution of aromatic, polar and positively charged residues were high while hydrophobic amino acid residues were low. In HSP60 family (Fig 3D), the distribution of aromatic, charged (both positive and negative charged) and polar residues were low. In HSP70 (Fig 3E), aromatic residues, positively charged residues and polar residues were depleted while negatively charged residues and hydrophobic residues were enriched. In HSP90 (Fig 3F), aromatic content, negatively charged residues and polar residues were enriched while positively charged residues were not significant. In HSP100 family (Fig 3G), hydrophobic residues were enriched, aromatic content and polar residues were depleted and charged residues (positively as well as negatively charged) were not significant.

### Performance of SVM during Cross Validation

#### 1^st^ tier of Prediction

Using FFCV and discrete amino acid composition as SVM input, we were able to achieve 72.98% overall accuracy with MCC 0.34. When coupled amino acid composition was used as input, the overall accuracy increased to 76.66% while MCC rose to 0.43 (Table 3). The result clearly shows that coupled amino acid composition based model performed better than discrete amino acid composition based model.

**Table 3:** Performance of discrete amino acid and coupled amino acid composition based SVM models during FFCV at 1^st^ tier.

Discrete Amino Acid Composition						Coupled Amino Acid Composition					
Sens	Spec	Accu	MCC	AUC	Para	Sens	Spec	Accu	MCC	AUC	Para
66.69	74.39	72.98	0.34	0.77	-z c -j 5 -t 2 -g 0.01	74.45	77.17	76.66	0.43	0.84	-z c -j 7 -t 1 -d 2

Sens, Spec, Accu, MCC, AUC and Para represents sensitivity, specificity, accuracy, Matthew’s correlation coefficient, area under ROC curve and SVM_light learning parameters on which performance was achieved respectively. All values except MCC and AUC are expressed in percentage.

#### 2^nd^ tier of Prediction

At 2^nd^ tier, the prediction was done to identify the family to which an HSP (predicted as 1^st^ tier) might belong. Similar to the 1^st^ tier, coupled amino acid composition based SVM model achieved higher accuracy than discrete amino acid composition in each family (Table 4).

**Table 4:** Performance of discrete amino acid and coupled amino acid composition based SVM models during LOOCV at 2^nd^ tier.

HSP Family	Discrete Amino Acid Composition						Coupled Amino Acid Composition					
	Sens	Spec	Accu	MCC	AUC	Para	Sens	Spec	Accu	MCC	AUC	Para
HSP20	84.87	86.24	86.02	0.60	0.96	-z c–j 7 t 2 –g 0.005	92.16	97.16	96.36	0.87	1.00	-z c -j 4 -t 2 -g 0.005
HSP40	86.55	84.88	85.84	0.71	0.94	-z c–j 1 t 1 –d 4	96.09	86.26	91.91	0.83	0.99	-z c -j 1 -t 2 -g 0.0005
HSP60	84.05	85.65	85.53	0.46	0.95	-z c–j 9 t 1 -d 5	79.75	97.24	95.96	0.72	1.00	-z c -j 10 -t 1 -d 3
HSP70	84.81	83.73	83.87	0.53	0.92	-z c–j 5 t 1 -d 5	91.17	91.97	91.87	0.71	1.00	-z c -j 6 -t 1 -d 2
HSP90	82.76	83.25	83.24	0.27	0.92	-z c–j 22 t 2 –g 0.0005	72.41	99.12	98.43	0.70	1.00	-z c -j 20 -t 2 -g 0.0005
HSP100	88.24	89.02	88.99	0.43	0.97	-z c–j 37 t 1 –d 5	82.35	98.08	97.48	0.71	1.00	-z c -j 19 -t 2 -g 0.0005

Sens, Spec, Accu, MCC, AUC and Para stand for sensitivity, specificity, accuracy, Matthew’s correlation coefficient, area under ROC curve and SVM_light parameter respectively. All values except MCC and AUC are expressed in percentage.

#### Receiver Operating Characteristics Curve Analysis

**Fig 4:**
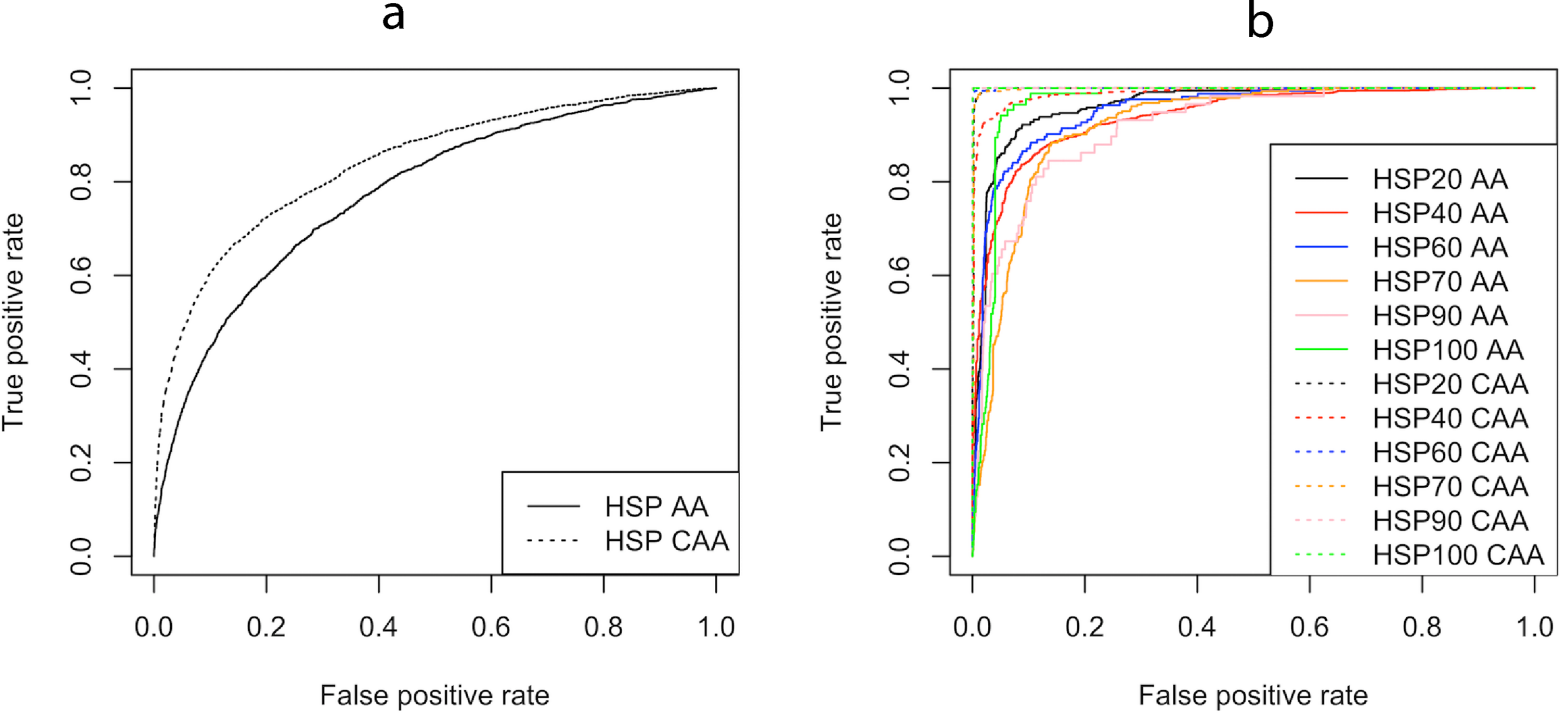
**ROC curve of SVM models based on amino acid and coupled amino acid composition for prediction of (4a) HSPs and (4b) different families of HSPs.** Solid line represents discrete amino acid composition (AA) while broken represents coupled amino acid composition (CAA) based SVM model.

Receiver operating characteristics (ROC) curve is a plot between sensitivity and false positive rate [34]. It shows the trade-off between sensitivity and specificity and can be used as a measure to assess the performance of a classifier. The area under the ROC curve is called AUC value [35], which quantifies the performance of the classifier. Higher AUC value shows better prediction. If AUC value reaches 1, it shows perfect prediction. We used ROCR package [36] to plot ROC curves and to calculate AUC values. ROC curve and AUC values of tier 1 and tier 2 SVM models also suggested that coupled amino acid composition was a better choice over the discrete amino acid composition (Fig 4, Table 4). Hence in further work, we used coupled amino acid composition based SVM models for the prediction of HSP and its families and termed it as predHSP.

### Comparative Performance *vis-à-vis* Existing Methods

It is important to compare the performance of a newly developed prediction method *vis-à-vis* the existing one. The method developed by Ahmad et al. [12] does not provide any family wise performance of HSP class prediction. So we compared the performance of PredHSP only with the method developed by Feng et al. and which was named as iHSP-PseRAAAC [1]. It was developed by using the 2,225 HSPs and the reduced amino acid composition as the input to classify a query protein into one of the six families of HSPs. In their paper, Feng et al. [1] described performance of five different types of reduced amino acid compositions namely (CP(13), CP(11), CP(9), CP(8) and CP(5)). Among all five modes, CP(11) was reported to have maximum performance. Hence we have compared performance of PredHSP with the performance of model developed using CP(11). We were able to compare our results for 2^nd^ tier SVM models only because iHSP-PseRAAAC only reported classification performance of six families as it was not intended to differentiate between HSP and non-HSP sequences.

Table 5 shows the jackknife success rate of identification in iHSP-PseRAAAC and PredHSP. The comparison clearly shows that the performance of PredHSP is better than iHSP-PseRAAAC both in terms of sensitivity and specificity. The higher success rate of PredHSP also shows that coupled amino acid composition encapsulates protein sequence attributes better than the simple/discrete as well as reduced amino acid composition.

**Table 5:** Comparison of performance of PredHSP with iHSP-PseRAAAC at 2^nd^ tier.

HSP Family	iHSP-PseRAAAC/PredHSP		
	Sensitivity	Specificity	MCC
HSP20	87.68/92.16	96.36/97.16	0.82/0.87
HSP40	95.31/96.09	84.87/86.26	0.99/0.83
HSP60	66.87/79.75	98.93/97.24	0.69/0.72
HSP70	79.15/91.17	86.54/91.97	0.54/0.71
HSP90	51.72/72.41	99.89/99.12	0.30/0.70
HSP100	69.41/82.35	99.84/98.08	0.83/0.71

There are two additional advantages of PredHSP over iHSP-PseRAAAC (i) unlike iHSP-PseRAAAC, PredHSP does not necessarily require only known HSP as query as it is capable to discriminate between HSPs and non-HSPs with very high accuracy and (ii) PredHSP has shown better performance than iHSP-PseRAAAC. It is anticipated that PredHSP become a useful high throughput tool in speeding up identification and classification of heat shock proteins.

### Performance of PredHSP on Independent Datasets

We also benchmarked the performance of PredHSP on two different datasets belonging to human (HGNC dataset) and rice (mixed dataset) respectively. In human HSPs, among 11 proteins of HSP20, PredHSP predicted only 2 as non-HSP and 1 HSP20 protein was classified to a wrong family (HSP40). Out of 49 proteins of HSP40 belonging to human, PredHSP predicted only 4 as non-HSP hence there were no misclassification. Among 14 HSP60 proteins, PredHSP predicted 4 HSPs as non-HSPs while 1 was predicted in wrong family (HSP70). For other two HSPs i.e., HSP70 and HSP90, there was no wrong prediction.

The proteins of different families of rice HSPs were obtained from [15] and [16]. Out of 14 HSP20, PredHSP predicted only 2 proteins as non-HSPs, while for HSP60, HSP70, HSP90 and HSP100, PredHSP did not give any false prediction (Table 6). PredHSP gave 23 true prediction as HSP70 while only one protein was misclassified as HSP20 from the proteins obtained from Sarkar et al [16].

**Table 6:** Performance of PredHSP on human HSPs obtained from HGNC [14] and rice HSPs obtained from Wang et al. [15] and Sarkar et al. [16]. TP represents true prediction and FP represents false prediction.

Source→	Human			Rice					
HSP	HGNC Database			Wang et al.			Sarkar et al.		
Class	Total	TP	FP	Total	TP	FP	Total	TP	FP
HSP20	11	8	3 (2-non-HSP, 1-HSP40)	14	12	2 (non-HSP)	—	—	—
HSP40	49	45	4 (non-HSP)	—	—	—	—	—	—
HSP60	14	9	5 (4 non-HSP, 1-HSP70)	4	4	0	—	—	—
HSP70	17	17	0	7	7	0	24	23	1 (HSP20)
HSP90	4	4	0	3	3	0	—	—	—
HSP100	—	—	—	3	3	0		—	—
**Total**	**95**	**83**	**12**	**31**	**29**	**2**	**24**	**23**	**1**

### Genome Wide Identification of HSPs

Since HSPs are present in all the three domains of life, thus we selected nine different proteome from archaea, prokaryotes and eukaryotes for annotation. We found 43 HSPs in *M*. thermautotrophicus, 51 in *E*. *coli*, 123 in *M*. *tuberculosis*, 145 in *S*. *cerevisiae*, 814 in *A*. *thaliana*, 2192 in *O*. *sativa*, 556 in *C*. *elegans*, 331 in *D*. *melanogaster* and 979 in *H*. *sapiens* (Table 7). The results clearly show that both plant species included in our study i.e., *Arabidopsis* and *Oryza* contains higher percentage of HSPs than other organisms which might be due the fact that plants tolerate extra abiotic stresses such as heat, drought, salinity, chemical toxicity, extreme temperature, oxidative stress and biotic stresses such as pathogen infection, insect attacks and other human activities [37, 38] etc. due to their immobile nature.

**Table 7:** Genome wide annotation of heat shock proteins in different organisms.

Organism	Total number of HSP	HSP20	HSP40	HSP60	HSP70	HSP90	HSP100
*M*. *thermautotrophicus (*1868*)*	43	8	9	5	13	1	7
*E*. *coli (*4305*)*	51	8	22	3	15	2	1
*M*. *tuberculosis (*3993*)*	123	15	42	5	42	2	17
*S*. *cerevisiae (*6721*)*	145	12	82	9	30	6	6
*A*. *thaliana (*31480*)*	814	137	406	70	149	19	33
*O*. *sativa (*37386*)*	2192	324	1212	158	403	11	84
*C*. *elegans (*26612*)*	556	94	252	54	125	13	18
*D*. *melanogaster (*22006*)*	331	62	172	24	61	4	8
*H*. *sapiens (*70076*)*	979	225	539	57	113	16	29

## Webserver

We have also established a webserver for the use of PredHSP by scientific community. It is freely available at http://14.139.227.92/mkumar/predhsp/index.html. A standalone version of PredHSP is also available at the above-mentioned link, which can be used to handle large data.

## Conclusions

HSPs are one of the largest groups of chaperones, which play a key role in protein folding and unfolding. In this work, we reported a SVM based two-tier prediction method, PredHSP, to identify HSPs and their families namely HSP20, HSP40, HSP60, HSP70, HSP90, and HSP100. Discrete amino acid composition and coupled amino acid composition were used as SVM input, however the later (check spelling) performed better at both levels. This may be due to the fact that discrete amino acid composition does not have the sequence order information. Performance results show that PredHSP is more efficient than the existing HSP classifier, iHSP-PseRAAAC. It is anticipated that PredHSP would be useful for high throughput prediction of HSPs prediction and would aid in basic research as well as in drug development.
